# Supplementary material for: Observation of the photonic Hall effect and photonic magnetoresistance in random lasers
Source: Nat Commun. 2024 May 30;15:4589. doi: 10.1038/s41467-024-48942-6 (PMC11139889; doi:10.1038/s41467-024-48942-6)
Supplement: Supplementary file 1 — Supplementary information [file 41467_2024_48942_MOESM1_ESM.pdf]

Supporting Information for

Observation of the photonic Hall effect and photonic magnetoresistance in random lasers

Wenyu Du[1,2,3], Lei Hu[1,2], Jiangying Xia[1,2], Lin Zhang[4], Siqi Li[1,2,3], Yan Kuai[1,2], Zhigang Cao[1,2,3], Feng Xu[2,3], Yu Liu[1,2,3], Kaiming Zhou[4], Kang Xie[5], Benli Yu[1,2,3], Ernesto P. Raposo[6], Anderson S. L. Gomes[7], Zhijia Hu[1,2,3]*

1 Information Materials and Intelligent Sensing Laboratory of Anhui Province, Anhui University, Hefei 230601, China

2 Key Laboratory of Opto-Electronic Information Acquisition and Manipulation of Ministry of Education, Anhui University, Hefei 230601, China

3 School of Physics and Opto-electronics Engineering, Anhui University, Hefei 230601, China

4 Aston Institute of Photonic Technologies, Aston University, Birmingham B4 7ET, UK

5 School of Opto-Electronic Engineering, Zaozhuang University, Zaozhuang 277160, Shandong, China

6 Laboratório de Física Teórica e Computacional, Departamento de Física, Universidade Federal de Pernambuco, 50670-901, Recife, PE, Brazil

7 Departamento de Física, Universidade Federal de Pernambuco, 50670-901, Recife, PE, Brazil

[*] Corresponding author E-mail: zhijiahu@ahu.edu.cn

Contents

Supplementary Information I:

The preparation of materials and fabrication process for the magnetic gain POF

Supplementary Information II:

The experimental setup

Supplementary Information III:

Photonic magnetoresistance in polymer fiber RLs: field-dependent RL spectroscopy

Supplementary Information IV:

Overlap distributions signalizing the photonic Hall effect and field-dependent RSB transition

Supplementary Information V:

Correlation coefficient heatmaps

Supplementary Information VI:

Simulation results based on scattering theory

References

Supplementary Information I

The preparation of materials and the fabrication process for the magnetic gain POF

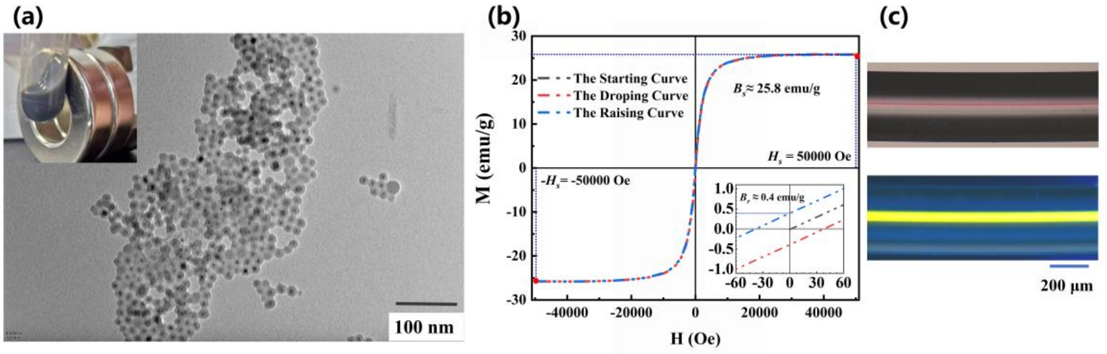

Figure S1. Magnetic gain POF fabrication and property characterization. (a) TEM image of Fe_3O_4@SiO_2 NPs (black scale bar, 100 nm), inset: dynamic variation of the Fe_3O_4@SiO_2 NPs under magnetic field. (b) Magnetic hysteresis loop at 300 K for Fe_3O_4@SiO_2 NPs, showing remnant magnetizations of 0.4 emu/g. The saturation magnetic field intensity is 50000 oe, the saturation magnetic induction intensity is 25.8 emu/g (Quantum Design, MPMS3). (c) Optical microscopic images illuminated by an incandescent lamp (above) and ultraviolet lamp (below) (blue scale bar, 200 μm).

In order to explore the PHE and PMR in RL, laser dye and magnetic Fe_3O_4@SiO_2 nanoparticles (NPs) are doped inside the fiber core. Figure S1(a) shows the TEM image of the NPs and the inset shows their attraction by an applied magnetic field. The magnetic hysteresis loop of these paramagnetic NPs is presented in Fig. S1(b). Figure S2 shows the energy spectrum of Fe_3O_4@SiO_2 NPs. The inset shows the content of each element. The magnetic gain polymer optical fiber (MGPOF) is produced via the prefabricated rod stretching method, described in [1]. First, a mold is assembled for the fiber optic prefabricated rod with a transparent Teflon tube with a diameter of 2 cm inserted into both ends of a homemade base, and a Teflon rope with a diameter of 0.8 mm is fixed at the center of the Teflon tube. Dilauroyl peroxide (LPO) with a mass fraction of 0.22% was added to the beaker as the initiator, followed by methyl methacrylate (MMA) and butyl acrylate (BA) in a volume ratio of 80:20, and n-butyl mercaptan as the chain transfer agent with a mass fraction of 0.18%. After ultrasonic

mixing, the solution is injected into the mold with a syringe as the cladding solution for the prefabricated rods, which are slowly heated to polymerize, and then the Teflon rope is drawn off to form a hollow prefabricated rod. Using vacuum suction, the core solution is drawn into the air holes in the middle of the preform, consisting of MMA and BA in a volume ratio of 85:15, LPO and n-butyl mercaptan in the same ratio as the cladding, laser dye PM597 with a mass fraction of 0.14%, and magnetic scattering nanoparticles (NPs) with a mass fraction of 0.3%. The MGPOF is a structure of NPs packaged in a polymer matrix, which forms a solid structure when polymerization completed. Referring to the method in the literature [2], core-shell structured $Fe_3O_4@SiO_2$ magnetic NPs are fabricated. The preform is again polymerized by slowly heating them up and placed on the fiber drawing tower for melt stretching to obtain the MGPOF. The refractive index of the cladding is 1.4882 and the refractive index of the core is 1.4903. Figure S1(d) displays two microscope photographs of the MGPOF, illuminated by incandescent (above) and UV lamps (bottom), with a cladding diameter of 674 μm (±0.5 μm) and a core diameter of 32 μm (±0.5 μm). The length of the MGPOF is 5 cm, which is consistent with the diameter of the magnetic pole, so the whole MGPOF-RL is placed in the magnetic field environment.

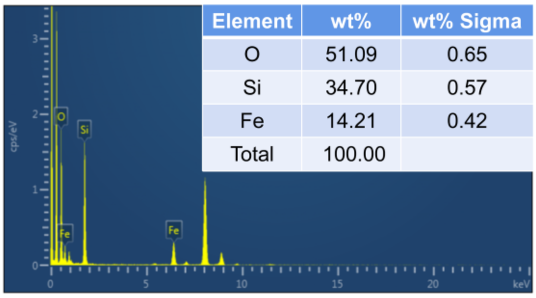

Figure S2. The energy spectrum of $Fe_3O_4@SiO_2$ NPs. Inset: the content of each element.

Supplementary Information II

The experimental setup

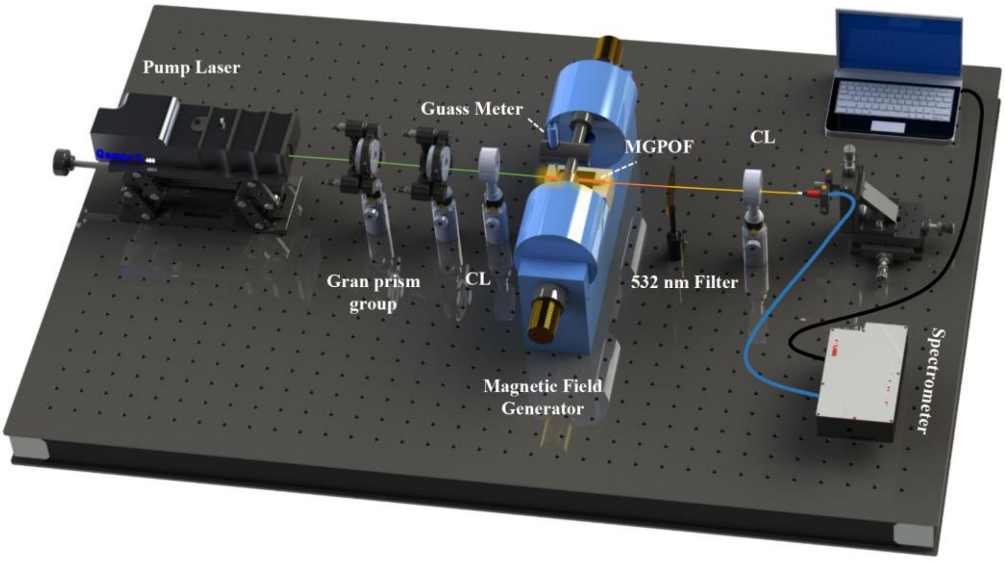

Figure S3. Experimental setup for measuring RL spectra of MGPOF. CL: convergent lens. Inset: MGPOF under laser excitation at a wavelength of 532 nm.

Supplementary Information III

Photonic magnetoresistance in polymer fiber RLs: field-dependent RL spectroscopy

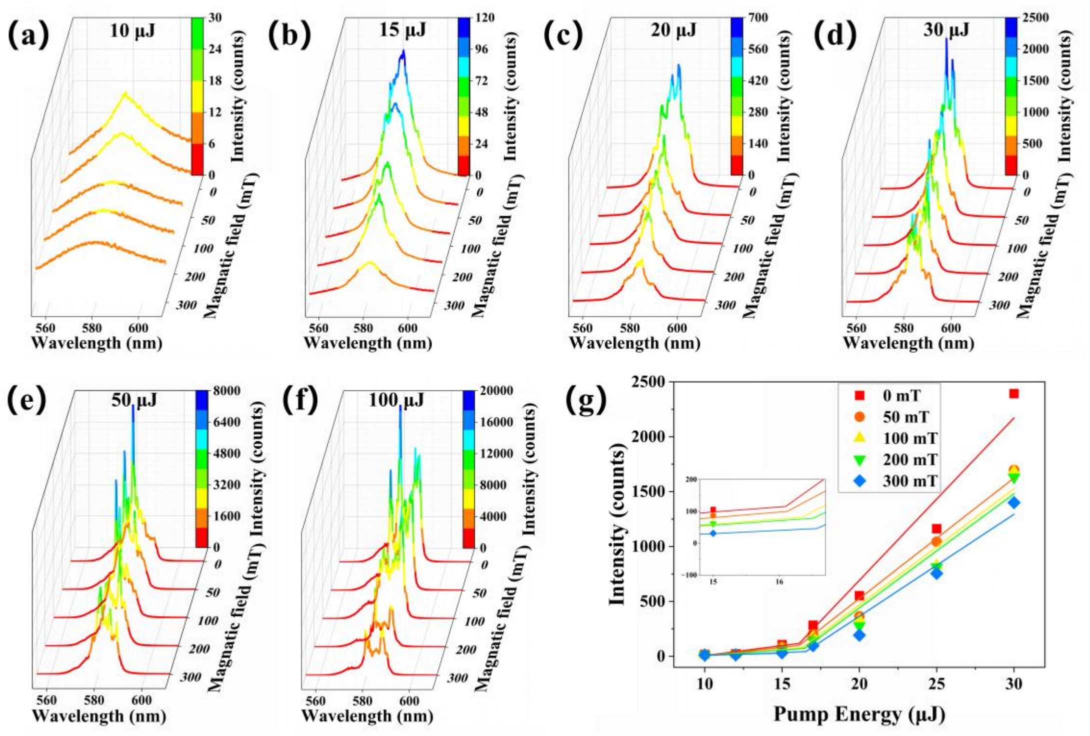

Figure S4. RL spectra at the applied magnetic field intensity of 0, 50, 100, 200, and 300 mT. Pump energy of 10 μJ for (a), 15 μJ for (b), 20 μJ for (c), 30 μJ for (d), 50 μJ for (e), and 100 μJ for (f). (g) Variation of the peak intensity of RL emission with pump energy. The inflection point of the fitted polyline corresponds to the threshold of the RL. Inset: The magnification of the inflection point.

Considering the modulation of the scattering particle dielectric constant by the magnetic field, the relative permittivity of the medium in the presence of light absorption is expressed as a complex number, $\varepsilon(\omega)=\varepsilon_1(\omega)+i\varepsilon_2(\omega)$, with $\varepsilon_1(\omega)$ and $\varepsilon_2(\omega)$ representing its real and imaginary parts, respectively. The refractive index of an absorbing medium is represented by $N(\omega)=n(\omega)+iK(\omega)$. The real part is denoted as

$n(\omega)$, which is commonly referred to as the refractive index, while the imaginary part $K(\omega)$ represents the extinction coefficient and is tied to material absorption. The light absorption coefficient is $\alpha(\omega)=2\omega K(\omega)/c$ and $[N(\omega)]^2=\varepsilon(\omega)$. Based on the above reasoning, it can be inferred that $\alpha(\omega)=\omega\varepsilon_2(\omega)/cn(\omega)$. When the imaginary part of the dielectric function causes the absorption of electromagnetic waves, the optical absorption coefficient is proportional to the imaginary part of the relative dielectric function. An increase in the dielectric constant caused by the magnetic field leads to a corresponding increase in absorption, resulting in a decrease in RL emission intensity.

Supplementary Information IV

Overlap distributions signalizing the photonic Hall effect and field-dependent RSB transition

We show in Fig. 4(f-o) and Fig. S5 (a-e) the distributions $P(q)$ of values of Parisi overlap parameter q for several excitation energies and magnetic field values. The maximum value of $P(q)$, denoted as $P(q)_{max}$, can be also used to describe the dispersion degree of the distribution. For Gaussian distribution, the standard deviation represents the degree of dispersion of the data around the mean, where a larger value indicates a wider distribution. From the shape of the Gaussian distribution curve, the larger the standard deviation, the flatter and wider the single-peaked curve. Conversely, when the standard deviation is smaller, the curve of Gaussian distribution will gather around the mean, and the higher and narrower the single-peaked curve will be. We count the sum of the extreme values of the distribution $P(q)$ when q_{ab} is positive and negative as Eq. (7) of the main text.

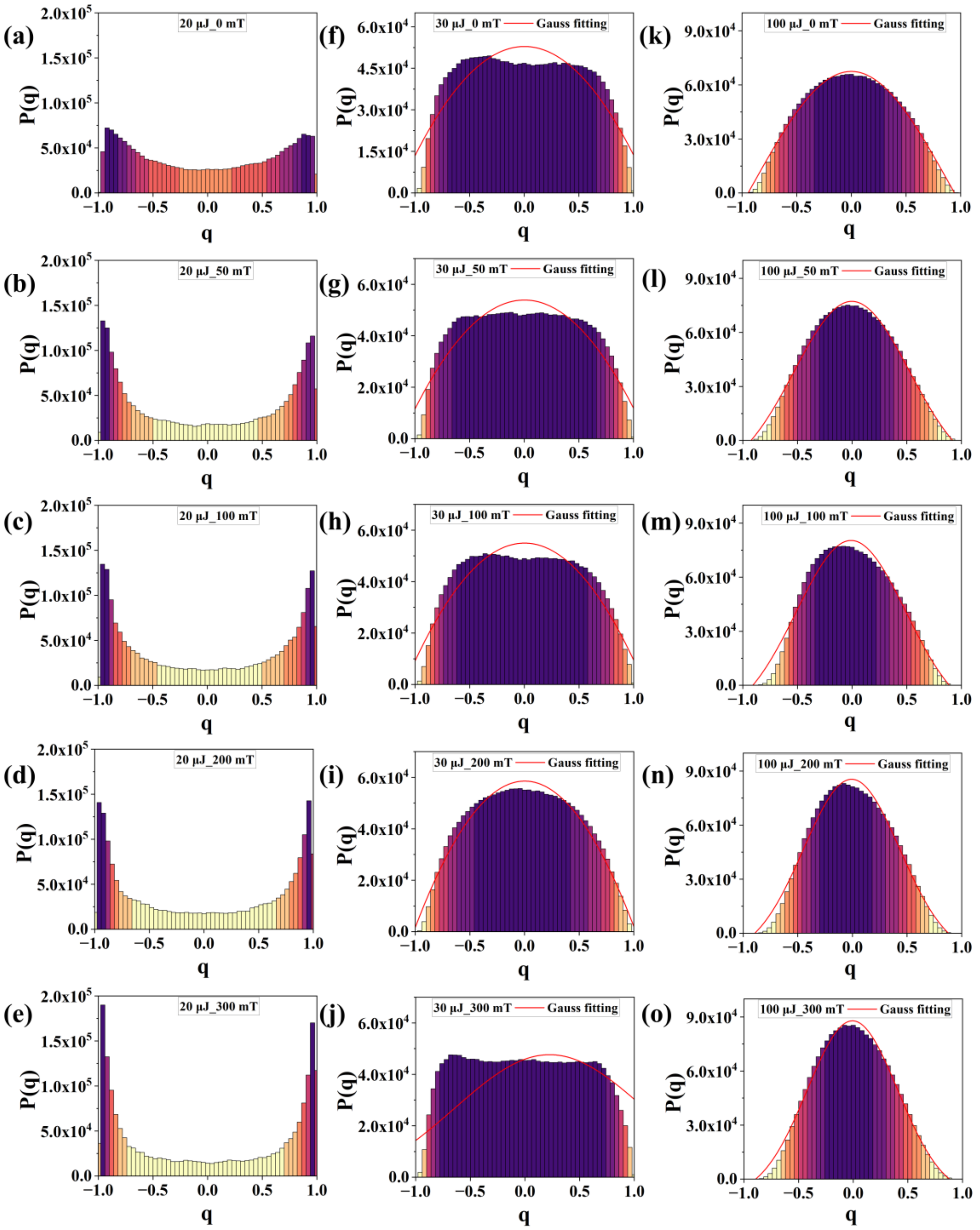

Figure S5. Overlap distributions signalizing the photonic replica-symmetic paramagnetic to RSB glassy transition. Plots of the distribution *P(q)* of the Parisi overlap parameter *q* for different pumping energies: 20 μJ: (a - e); 30 μJ: (f - j); 100 μJ: (k - o); at applied magnetic field strength of 0 mT (the first row), 50 mT (the second row), 100 mT (the third row) 200 mT (the fourth row) and 300 mT (the fifth row).

Supplementary Information V

Correlation coefficient heatmaps

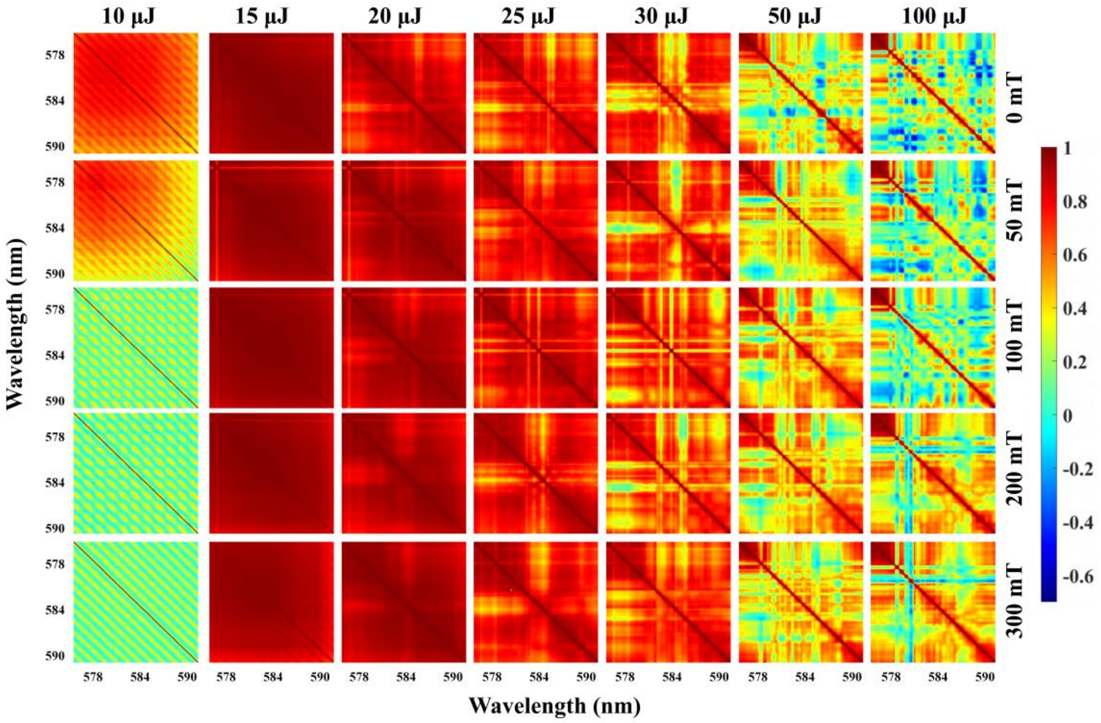

Figure S6. Correlation coefficient heatmaps of the spectral intensities across the RL quasimodes at the pump energy of 10 μJ (the first column), 15 μJ (the second column), 20 μJ (the third column), 25 μJ (the fourth column), 30 μJ (the fifth column), 50 μJ (the sixth column) 100 μJ (the seventh column); at applied magnetic field strength of 0 mT(the first row), 50 mT(the second row), 100 mT(the third row), 200 mT(the fourth row) and 300 mT(the fifth row).

We define the correlation coefficient between distinct wavelengths in the same spectrum as

$$r(\lambda_1,\lambda_2)=\frac{c(\lambda_1,\lambda_2)}{\sqrt{\upsilon[\lambda_1]\upsilon[\lambda_2]}}. \qquad \text{(S1).}$$

In the above equation, $\upsilon[\lambda_1]$ and $\upsilon[\lambda_2]$ are the variances of the RL intensity fluctuations at wavelengths λ_1 and λ_2, respectively, and c is their covariance. The correlation coefficient plots presented in Fig. S6 serve to quantify the correlation between RL modes intensities across all replicas. The correlation coefficients are visually displayed

in a heat map format, whereby diagonal elements delineate the autocorrelation of individual modes, and off-diagonal elements represent the intercorrelation between distinct modes. Positive values denote positive correlations either among the modes themselves or between different modes, whereas negative values indicate negative correlations.

The correlation coefficient thermal plot obtained at a 10 μJ pumping energy below the threshold indicates an enhancement of positive correlations with increasing magnetic field levels. Notably, at this pump energy level, no RL modes are present. Figure S4(a) shows some small spikes appearing in the absence of a magnetic field. However, these spikes are suppressed upon introducing an applied magnetic field, leading to fluorescence generation through spontaneous radiation. When the applied magnetic field strength is 0 and 50 mT, these orange thermograms with $r \approx 0.6$ indicate some correlation between spikes, while most of the green and yellow thermograms with $r \approx 0$ indicate almost irrelevant intensity fluctuations between the wavelengths of the fluorescence. Subsequently, high pumping power induces the onset of wavelength correlation as depicted by orange dots with $r \approx 0.6$ observed within the yellow thermograms with $r \approx 0$. These clear diagonal lines mark the autocorrelation between the different modes and correspond to the Gaussian-like distributions of $P(q)$, indicating the photonic paramagnetic state. These somewhat periodic off-diagonal stripes appear only below and around the threshold, possibly because the wavelengths of fluorescence emission are more stable with each other. The competition for gain in the fluorescent regime is at a comparable level and, as the system progressively enters the RL regime above threshold, the random character of the nonlinear couplings between field amplitudes becomes increasingly relevant. Interestingly, a similar phenomenon has been also reported below and around the threshold in other RL and random fiber laser systems [3-5].

There are near-full red thermograms with $r \approx 1$ observed in the vicinity of the 15 μJ pump, signaling a robust interrelation across the modes, corresponding to the spin glass phase distribution portrayed in Fig. 4(f-j). While the diagonal elements in the thermogram are very indistinct, which indicates the uncorrelation between the RL intensity fluctuations at each wavelength near the threshold. As the applied magnetic field increases, the diagonal line becomes more pronounced, showing that the magnetic

field enhances the correlation of the RL intensity fluctuations and attenuates the scattering disorder. 20 μJ presents similar correlation coefficient results.

When the pump energy is raised to 25 μJ, the clear horizontal and vertical lines indicate the cross-correlation between different modes corresponding to the paramagnetic to glassy transitions portrayed in Fig. 4(k-o). Unlike the uniform distribution of thermogram colors at 15 μJ, the cross-correlation distribution is discrete. The quasi-mode competition between RLs strengthens during this period, correlating with the sharp RL spikes found in Fig. S3(c). The red region with $r \approx 1$ expands as the magnetic field grows, implying that the magnetic field enhances inter-mode correlation while mitigating scattering disorder. 30 μJ exhibits similar correlation coefficient results.

At the 50 μJ pumping power level, a negative blue value with $r < 0$ appears in the correlation thermogram, indicating a negative correlation between intensity fluctuations at certain wavelengths of the RL at higher pump energy levels. Certain RL quasi-modes clearly overwhelm others in mode competition demonstrated by the single highest RL emission peak in Fig. S4(e). The RL intensity fluctuations at this point revert to a Gaussian-like distributed statistical state, indicating that the RL reverts to the paramagnetic phase where some modes dominate. As the magnetic field increases, the area of negative blue islands reduces. This suggests that the magnetic field mitigates the negative correlation between RL quasi-modes, while enhancing scattering and mode competition order. 100 μJ exhibits similar correlation coefficient results. We confirm the above analysis with simulation results based on scattering theory in Supplementary Information VI.

Supplementary Information VI

Simulation results based on scattering theory

We probe the findings and analysis of this work with numerical simulation results based on scattering theory. The scattering coefficient K_{sca}, extinction coefficient K_{ext}, and absorption coefficient K_{abs} [6] are

$$\begin{aligned} k_{\mathrm{sca}} &= \frac{2}{\alpha^2}\sum_{n=1}^{\infty}(2n+1)\left(|a_n|^2+|b_n|^2\right) \\ k_{\mathrm{ext}} &= \frac{2}{\alpha^2}\sum_{n=1}^{\infty}(2n+1)\mathrm{Re}\left(a_n+b_n\right) \\ k_{\mathrm{abs}} &= k_{\mathrm{ext}}-k_{\mathrm{sca}} \end{aligned} \quad \text{(S2),}$$

where α is the particle size parameter $\alpha=\pi d/\lambda$. In the above equation, d is the diameter of the particle and λ is the wavelength.

$$\begin{aligned} a_n &= \frac{\mu_{\mathrm{r}}\psi_n(\alpha)\psi_n'(\beta)-m\psi_n'(\alpha)\psi_n(\beta)}{\mu_{\mathrm{r}}\zeta_n^{(1)}(\alpha)\psi_n'(\beta)-m\zeta_n^{(1)'}(\alpha)\psi_n(\beta)} \\ b_n &= \frac{m\psi_n(\alpha)\psi_n'(\beta)-\mu_{\mathrm{r}}\psi_n'(\alpha)\psi_n(\beta)}{m\zeta_n^{(1)}(\alpha)\psi_n'(\beta)-\mu_{\mathrm{r}}\zeta_n^{(1)'}(\alpha)\psi_n(\beta)} \end{aligned} \quad \text{(S3),}$$

In the above equation, $\beta=m\alpha$. m is the relative refractive index, $m=\sqrt{\varepsilon_{\mathrm{r}}\mu_r}$. ε_{r} is the relative permittivity and μ_r is the relative magnetic permeability. Meanwhile $m=n\text{-}i\eta$. The imaginary part η characterizes the absorption of the medium particle. ψ_n and ζ_n satisfy the following recurrence relations [7]:

$$\begin{aligned} \psi_n(Z) &= \frac{2n-1}{Z}\psi_{n-1}(Z)-\psi_{n-2}(Z) \\ \psi'_n(Z) &= -\frac{n}{Z}\psi_n(Z)+\psi_{n-1}(Z) \\ \xi_n(Z) &= \frac{2n-1}{Z}\xi_{n-1}(Z)-\xi_{n-2}(Z) \\ \xi_n'(Z) &= -\frac{n}{Z}\xi_n(Z)+\xi_{n-1}(Z) \end{aligned} \quad \text{(S4).}$$

ψ_n and ζ_n also satisfy the following initial conditions:

$$\begin{aligned}
&\psi_{-1}(Z) = \cos Z \\
&\psi_{0}(Z) = \sin Z \\
&\xi_{-1}(Z) = \cos Z - i \sin Z \\
&\xi_{0}(Z) = \sin Z + i \cos Z
\end{aligned} \tag{S5}$$

The slope of the M-H curve decreases with increasing magnetic field strength H as shown in Fig. S1(b). The slope of the M-H curve is proportional to the permeability μ_r, *i.e.*, as H increases, μ_r decreases. As in Fig. S7 (a-b), K_{sca} decreases as μ_r decreases. Moreover, $K_{sca}=\sigma_{sc}/S_p$, where S_p is the projected area of the scatter against the direction of light propagation. From the above, it can be concluded that

$$k_{\text{sca}} = \frac{1}{\rho S_p l_s} \tag{S6}$$

A decrease in K_{sca} causes an increase in l_s. In Fig. S7(c-f), K_{abs} and K_{ext} increase as μ_r decreases. When μ_r decreases with increasing H, K_{sca} decreases, which in turn causes the decrease in σ_{sc} and the increase in l_s. The reduced σ_{sc} reduces the overall scattering disorder, which in turn reduces the scattering disorder of the RL. This demonstrates that the magnetic field increases the orderliness of the RL disorder scattering, which verifies the PHE in RLs. The increase in l_s confirms the previous conclusion that the material absorption increases when the magnetic field increases, and reduces the RL intensity, which verifies the PMR in RLs. Overall, the simulation results are in good agreement with the experimental results.

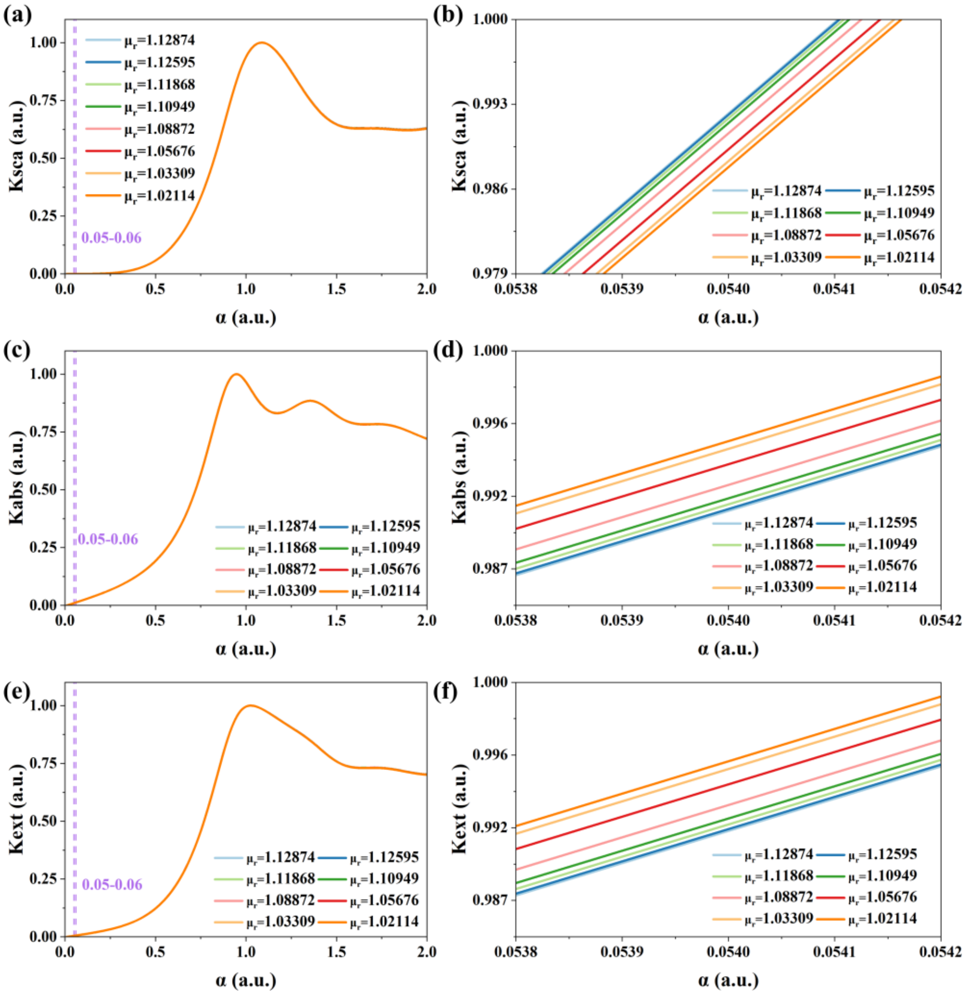

Figure S7. Simulation results of (a) K_{sca}, (c) $K_{ext,}$ and (e) K_{abs} varying with μ_r. Purple dotted line: α=0.05~0.06 (λ=530nm~560nm). (b), (d) and (f) are the enlarged curves at the purple dotted line.

References

1. Z. J. Hu, Y. Y. Liang, P. F. Gao, H. M. Jiang, J. J. Chen, S. H. Jiang and K. Xie, "Random lasing from dye doped polymer optical fiber containing gold nanoparticles," Journal of Optics, 17, 125403 (2015).

2. J. Lee, Y. Lee, J. K. Youn, H. Bin Na, T. Yu, H. Kim, S. M. Lee, Y. M. Koo, J. H. Kwak, H. G. Park, H. N. Chang, M. Hwang, J. G. Park, J. Kim, and T. Hyeon, "Simple synthesis of functionalized superparamagnetic magnetite/silica core/shell nanoparticles and their application as magnetically separable high-performance biocatalysts," Small 4, 143-152 (2008).

3. E. Coronel, A. Das, I. R. R. González, A. S. L. Gomes, W. Margulis, J. P. von der Weid, and E. P. Raposo, "Evaluation of Pearson correlation coefficient and Parisi parameter of replica symmetry breaking in a hybrid electronically addressable random fiber laser," Optics Express 29, 24422-24433 (2021).

4. E. D. Coronel, M. L. da Silva-Neto, A. L. Moura, I. R. R. González, R. S. Pugina, E. G. Hilário, E. G. da Rocha, J. M. A. Caiut, A. S. L. Gomes, and E. P. Raposo, “Simultaneous evaluation of intermittency effects, replica symmetry breaking and modes dynamics correlations in a Nd:YAG random laser, ”Scientific Reports 12, 1051 (2022).

5. E. D. Coronel, A. Das, M. L. da Silva-Neto, I. R. R. González, A. S. L. Gomes, and E. P. Raposo, “Statistical analysis of intensity fluctuations in the second harmonic of a multimode Nd:YAG laser through a modified Pearson correlation coefficient,” Physical Review A 106, 063515 (2022).

6. Hulst, Hendrik Christoffel van de. “Light scattering by small particles,” Johnwiley & Sons: New York, (1957).

7. W. J. Wiscombe, “Improved Mie scattering algorithms,” Appl. Optics 19, 1505-1509 (1980).
